# Correction: ‘Impact of infectious diseases on wild Bovidae populations in Thailand: insights from population modelling and disease dynamics’ (2024), by Horpiencharoen *et al*.

**DOI:** 10.1098/rsif.2024.0610

**Published:** 2024-10-30

**Authors:** Wantida Horpiencharoen, Jonathan C. Marshall, Renata L. Muylaert, Reju Sam John, David Hayman

**Affiliations:** ^1^ Hopkirk Research Institute, Massey University Molecular Epidemiology and Public Health Laboratory, Palmerston North, Manawatu-Wanganui, New Zealand

**Keywords:** bovine, disease transmission, prediction, population, wildlife conservation

*J. R. Soc. Interface* **21**: 2120240278 (Published online 03 July 2024). (http://doi.org/10.1098/rsif.2024.0278)

We have identified two errors in the manuscript and made the following corrections:

1. Correction of Pathogen Name:
  — *Mycobacterium tuberculosis* was used in a few places instead of *Mycobacterium bovis*. We have corrected this in:
    - In table 2: ‘Pathogens’ column.
    - In Subheading 2.1.4.2: The title has been corrected to ‘Bovine tuberculosis (*Mycobacterium bovis*)."
2. Equation Correction in table 1 Footnote:
  — We have corrected the equation in the footnote of table 1 by adding minus sign, changing the equations as follows:
    - $1-{exp}^{rt}$ to $1-{exp}^{-rt}$
    - $1-{exp}^{{(2x10}^{-5})*365}$ to $1-{exp}^{{-(2x10}^{-5})*365}$

